# The role of miR-711 in cardiac cells in response to oxidative stress and its biogenesis: a study on H9C2 cells

**DOI:** 10.1186/s11658-020-00206-z

**Published:** 2020-04-09

**Authors:** Duo Zhao, Hao Zheng, Adam Greasley, Fengjun Ling, Qinfeng Zhou, Bowen Wang, Tiffany Ni, Ishita Topiwala, Cuilin Zhu, Tina Mele, Kexiang Liu, Xiufen Zheng

**Affiliations:** 1 Department of Cardiovascular Surgery, The Second Hospital of Jilin University, Changchun, 130041 China; 2 Department of Pathology and Laboratory Medicine, Western University, London, Ontario Canada; 3 Department of Cardiovascular Surgery, The First People’s Hospital of Foshan, Foshan, Guangdong China; 4 Department of Laboratory Medicine, Zhangjiagang TCM Hospital Affiliated to Nanking University of Chinese Medicine, Zhangjiagang, Jiangsu China; 5 Department of Surgery, Western University, Ontario, London Canada; 6 London Health Sciences Centre, London, Ontario Canada; 7 Department of Oncology, Western University, Ontario, London Canada; 8 Lawson Health Research Institute, Ontario, London Canada

**Keywords:** miR-711, Oxidative stress, HIF-1α, NFКB, Ang-1, FGF14, Cacna1c

## Abstract

**Background:**

Oxidative stress results in cell apoptosis/death and plays a detrimental role in disease development and progression. Stressors alter the miRNA expression profile and miRNAs play a role in the cell response to stress. We previously showed that miR-711 is significantly over-expressed in extended cold ischemia reperfusion injured hearts in heart transplant. In this study, we aimed to investigate the role of miR-711 in cardiac cell damage in response to oxidative stress and how miR-711 is regulated.

**Methods:**

Rat cardiac cell line H9c2 cells were cultured and exposed to oxidative conditions (Antimycin A (AA), H_2_O_2_, CoCl_2_, or cold hypoxia/reoxygenation (H/R)) in vitro. H9c2 cells were transfected with miR-711 mimics, miR-711 inhibitors, or small interference RNA, using transfection reagents. The expression of miR-711 was measured by quantitative reverse transcriptase-polymerase chain reaction (qRT-PCR). Cell apoptosis/death was detected by flow cytometry and an IncuCyte system. Mitochondrial damage was detected by measuring the mitochondria membrane potential by flow cytometry. Gene expression was detected by qRT-PCR at the mRNA level and Western blotting and immunocytochemistry staining at the protein level.

**Results:**

We found that miR-711 was significantly up-regulated in cells treated with H_2_O_2_, AA, CoCl_2_, and cold H/R. Over-expression of miR-711 increased cell apoptosis/death induced by AA and H/R whereas cell death was reduced by miR-711 inhibitors. MiR-711 induced cell death through negative regulation of angiopoietin 1 (Ang-1), fibroblast growth factor 14 (FGF14) and calcium voltage-gated channel subunit alpha1C (Cacna1c) genes. Both knockdown of hypoxia inducible factor 1α (HIF-1α) and inactivation of the nuclear factor kappa-light-chain-enhancer of activated B cells (NFКB) pathway inhibited over-expression of miR-711.

**Conclusion:**

Oxidative stress increases the expression of miR-711. Over-expression of miR-711 induces cell apoptosis/death. HIF-1α and NFКB regulate miR-711 in H9c2 cells during oxidative stress. miR-711 is a new target for preventing oxidative stress.

## Background

Oxidative stress occurs when the balance between free radical oxygen species (ROS) and antioxidants is disturbed. Oxidative stress causes cell apoptosis/death and plays a detrimental role in the development and progression of diseases including cardiovascular disease, neurodegeneration diseases, cancer and aging. Ischemia reperfusion (I/R) or H/R during cardiovascular surgery and treatment of myocardial infarction can produce excessive ROS, causing oxidative stress, leading to cardiac cell damage. Reducing oxidative stress could help improve heart function and outcome of treatment. It is important to understand the molecular mechanisms underlying oxidative stress mediated damage and to identify new targets for reducing oxidative stress.

microRNAs (miRNA) are single-stranded RNAs 18–24 nucleotides in length, that are generated from an endogenous transcript that consists of a hairpin structure [1–4]. Studies have shown that miRNA can negatively function as a gene regulator at post-transcriptional level through binding to the seed regions of the 3′-untranslated region (3′UTR) of target genes, leading to translational repression [5–9]. By inhibiting expression of target genes, miRNAs play crucial roles in various cellular processes and disease development [10–14]. It is estimated that miRNAs regulate one third of all genes [15]. Deregulation of miRNAs is associated with different forms of heart diseases [14, 16–20]. We previously demonstrated that the expression of miRNAs is dysregulated in I/R injured heart cells and that miR-711 is significantly up-regulated with the greatest fold change [21]. Recent studies have shown that miR-711 is up-regulated in trauma injured brains and ischemic heart cells and that its over-expression causes cell death [22–25]. miR-711 is also involved in HIV infection [26] and cancer [27, 28]. It is currently unknown how miR-711 is regulated during oxidative stress and by what molecular mechanism miR-711 causes cell death in response to oxidative stress.

The aim of the present study is to investigate the impact of miR-711 in cardiac cells in response to oxidative stress and the mechanisms of miR-711 regulation, in order to find new targets for developing a therapeutic approach for preventing oxidative stress.

## Materials and methods

### Cell culture

H9c2 cells obtained from ATCC (Manassas, VA) were cultured in Dulbecco’s Modified Eagle’s medium (DMEM, Invitrogen, Burlington, ON) with 10% fetal bovine serum (FBS, Invitrogen, Burlington, ON, Canada) and 100 U/ml penicillin-streptomycin (Sigma-Aldrich, Oakville, ON, Canada) in a humidified atmosphere of 5% CO_2_ at 37 **°**C.

### Induction of oxidative stress

In order to induce oxidative stress, H9c2 cells were plated in a 6-well plate at a density of 10^5^ cells per well and grown at 37 **°**C for 24 h prior to starting the oxidative stress protocol. Cells were washed with phosphate buffered saline (PBS, Thermo Fisher Scientific, Mississauga, ON, Canada) and treated with 1 mL of AA (Sigma Aldrich) at various concentrations described in figure legends in Ca^++^/Mg^++^ PBS (Thermo Fisher Scientific) at 37 **°**C for 1–3 h. After treatment, the cells were washed with PBS and cultured in 1 mL of DMEM complete medium (DMEM-CM) supplemented with 10% FBS at 37 **°**C for 3 h. This is a chemical method of stimulating I/R (Dutta et al. 2013).

Alternatively, cells were treated with either H_2_O_2_ or CoCl_2_ at indicated concentrations for 6 h, 12 h or 24 h to induce oxidative injury.

### Induction of simulated cold ischemia-reperfusion injury in vitro

To induce simulated cold ischemia-reperfusion injury, an in vitro hypothermal H/R model was used. Cell culture medium of plated H9c2 cells was aspirated and cells were washed once using PBS. PBS was then added to each well (1 mL/well for 6-well plates; 100 μL/well for 96-well microplates) before plates were placed in the HypOxystation H35 (Don Whitley Scientific, Shipley, UK) set at 10 °C and chamber conditions of 0.5% O_2_, 4.0% CO_2_, and 95.5% N_2_ for 18 h. Cells were then removed from the HypOxystation H35 and complete DMEM medium with 10% FBS was added to each well (1 mL/well for 6-well plates; 100 μL/well for 96-well microplates). Cells were then incubated for 24 h at 37 °C and 5% CO_2_. Cells in the normoxia group were incubated at 37 °C and 5% CO_2_.

### Cell transfection

H9C2 cells were transfected with HIF-1α siRNA, GL2 siRNA (Thermo Fisher Scientific), miR-711 mimics, miR-711 inhibitors or their relative controls (Qiagen) using Lipofectamine 2000 transfection reagent (Thermo Fisher Scientific) or Endofectin (GeneCopoeia, Rockville, MD) to knock down HIF-1α and miR-711, or over-express miR-711. Cells were placed in a 6-well plate at a density of 10^5^ cells per well and allowed to grow at 37 **°**C for 24 h. 2 μL of Lipofectamine 2000 reagent or Endofectin was used to transfect 1 μg of siRNA or miR-711mimic, or the respective inhibitors, according to the manufacturer’s instructions. Four hours after transfection, 700 μL of DMEM with 20% FBS was added to each well and the cells were incubated at 37 **°**C for 24 h.

### RNA isolation

Cells were washed with PBS, then Trizol (Qiagen, Toronto, ON, Canada) was added to lyse cells to obtain RNA. Total RNA including miRNAs was extracted using the miRNeasy mini kit (Qiagen, Cat# 217004) or miRNeasy Micro Kit (Qiagen, Cat# 217084) according to the manufacturer’s protocol. The concentration of RNA was determined by a NanoDrop UV-Vis Spectrophotometer (Thermo Fisher Scientific).

### Quantitative reverse transcriptase-polymerase chain reaction (qRT-PCR)

cDNA was synthesized with 1 μg of total RNA using a miScript II RT Kit (Qiagen, Cat # 218161) according to the manufacturer’s protocol. miScript HiSpec buffer was used for detecting mature miRNA during cDNA synthesis, while miScript HiFlex buffer was used for detecting both mature and precursor miR-711. cDNA for gene expression was synthesized using olig-dT primers and reverse transcriptase (RT) (New England Biolabs, Quebec, Canada), following the instructions of the manufacturer.

Levels of miR-711 were measured by qRT-PCR using miScript SYBR Green PCR Kits (Qiagen, Cat# 218073) and primers (Qiagen, Cat# MS00017696) according to the manufacturer’s instructions. Briefly, qPCR was performed in a 25 μl reaction volume consisting of 12.5 μl of 2 x SYBR green master mix, 2.5 μl of 10 x universal primers (Qiagen, Cat# MS00000001) and 2.5 μl of 10x miR-711 primers or Hs_Snord 61_11 (Qiagen, Cat# MS00033705) and 1 μl of 1:10 diluted cDNA. Reactions were performed using the CFX Connect Real-Time PCR Detection System (Bio-Rad Laboratories Ltd., Mississauga, ON, Canada). PCR was heat shocked for 15 min, followed by 40 cycles of 95 °C for 5 s, 55 °C for 30 s and 72 °C for 30 s. Snord 61 was used as a loading control. Expression of miR-711 was calculated by the ∆∆Ct method.

Levels of HIF-1α and pre-miR-711 were measured using primers obtained from Invitrogen, Burlington, ON. The reaction mixture contained: 1x SYBR Green PCR Master Mix (Bioline, Canada), 0.2 μM forward and reverse primer mix, and 1 μL of 1:10 diluted cDNA. Reactions were performed in the CFX Connect Real-Time PCR Detection System. The following thermocycling conditions were used: initial denaturation at 95 °C for 3 min, and 40 cycles of denaturation at 95 °C for 10 s, annealing at 60 °C for 10 s and extension at 72 °C for 20 s. Data were standardized to the GAPDH housekeeping gene. Gene expression was calculated by the ∆∆Ct method.

### Bay 11–7082 and Ly 294002 treatment

H9C2 cells were cultured in DMEM-CM medium supplemented with 10%FBS overnight. Cells were washed with DMEM basal medium once and treated with 1 μM Bay 11–7082 (Sigma) or 5 μM Ly 294002 (Sigma) in 1 ml of DMEM basal medium at 37 °C for 1 h. Cells were washed with DMEM basal medium and cultured in DMEM-CM supplemented with 10% FBS.

### Annexin-V FITC labelling and propidium iodide (PI) staining

Cell apoptosis/death was detected using the Annexin-V Apoptosis Detection Kit (Biolegend, San Diego, CA) according to the manufacturer’s instruction. In brief, the centrifuged cells were suspended in 1 mL of 1 x Annexin-V binding buffer (Biolegend) and centrifuged at 300 x g and 4 °C for 5 min. Cells were resuspended in 100 μl of 1 x Annexin-V binding buffer. 3 μL of Annexin-V FITC and 5 μl of propidium iodide (PI) (Biolegend) were added to the cell suspension. The cells were incubated in the dark for 15 min at room temperature. Another 200 μl of 1x Annexin-V binding buffer was added onto stained cells. Cells were then analyzed by flow cytometry using a CytoFLEX system (Beckman Coulter, Mississauga, Ontario) and the CytExpert software.

### Dynamic detection of cell death using SYTOX green in an IncuCyte system

To detect cell death dynamically, an IncuCyte system was used. DMEM complete medium (1 mL/well for 6-well plates; 100 μL/well for 96-well microplates) containing 0.25 μM freshly diluted SYTOX Green Nucleic Acid Stain (Thermo Fisher Scientific) was added to cells during the simulated reperfusion stage. Cells were then placed in an IncuCyte ZOOM system (Essen Bioscience, Ann Arbor, MI), set at 37 °C and 5% CO_2_ and imaged every hour. Using the IncuCyte’s basic analyzer program, the number of green objects and the green fluorescence mean in each well were counted as a measure of cell death in each treatment group at the end of the 24 h.

### Western blotting

To detect gene expression at the protein level, Western blotting was performed. Total proteins were extracted with radio immunoprecipitation assay (RIPA) buffer containing protease inhibitor PMSF (Cell Signaling Technology, Whitby, ON, Canada) followed by 3 cycles of 5 s sonication. Cell lysate was centrifuged for 20 min at 15,000 rpm and supernatant was collected. The concentration of proteins was measured using the BCA assay with the Pierce BCA Protein Assay Kit (Thermo Fisher Scientific). 25 μg total protein was loaded on 10% (Cacna1c) or 12% (all other genes) polyacrylamide gels (Sigma) and run for 60 min–90 min at 100 V. Separated proteins were transferred to polyvinylidene difluoride (PVDF) membranes (Bio-Rad). Transferred membranes were blocked with 5% fat-free milk powder in Tris-buffered saline (TBS) and Tween 20 (TBST) for 30 min at room temperature and then blotted with the primary antibodies against mouse Angiopoietin 1 (Ang-1) (1:1000 dilution, Abcam, Cat# ab10516, Cambridge, MA), phosphorylated p65 (p-p65) (1:1000, Cell Signaling Technology, Cat# 3033S), FGF14 (1:1000 dilution, Sc-16,812, Santa Cruz Biotechnology, Dallas, TX), calcium voltage-gated channel subunit alpha1C (Cacna1c1, Abcam, Cat# ab84814), Bax (1:3000 dilution, Cell Signaling Technology, Cat# 14796S), caspase 3 (1:1000 dilution, Cell Signaling Technology, Cat# 14220S), and β-actin (1:4000 dilution, Santa Cruz Biotechnologies, Cat# sc-81,178) at 4 °C overnight. The blotted membranes were washed with TBST containing 0.25% Tween-20 for 10 min at room temperature and washed three times. Washed membranes were blotted with appropriate 2nd antibodies (Santa Cruz Biotechnologies) for 30 min at room temperature. Proteins were developed with ECL kits (Bio-Rad) and visualized by the FluorChem M system (ProteinSimple, San Jose, CA). The density of bands was semi-quantified by the ImageJ program.

### In situ immunocytochemical staining (ICC)

To detect protein expression in situ, ICC was conducted. Cells were fixed with 4% freshly prepared paraformaldehyde (PFA) for 10 min at room temperature, rinsed with 1 ml cold PBS twice and permeabilized with 0.25% Triton PBS for 10 min at room temperature. Cells were blocked with 0.1% Tween 3% BSA (or 5% normal goat serum) PBS at room temperature for 1 h, then cells were blotted with 1st antibody (1:100) diluted in 0.1% Tween 3% BSA at 4 °C overnight. Abs used were the same as for Western blotting. After washing with PBST (0.1% Tween) for 5 min three times, cells were blotted with 1:500 2nd Abs: Alexa Fluor 488 labelled donkey anti-goat IgG (Thermo Fisher Scientific, Cat# A11055), Alexa Fluor 488 chicken anti-rabbit IgG (Thermo Fisher Scientific, Cat# A-21441) or Alexa 594 labelled goat anti-mouse IgG (Thermo Fisher Scientific Cat# A-11032) for 1 h at room temperature, followed by washing with PBST for 5 min, 3 times at room temperature. Cells were then mounted with Prolong Diamond anti-fade mounting media containing DAPI. Cells were imaged under a fluorescent microscope (Olympus) at × 200 magnification.

### Luciferase reported assay

To confirm miR-711 binding sites, a Luciferase reported assay was conducted. PmirGLO Dual-Luciferase reporter plasmids were purchased from Promega (Madison, WI, USA). Dual-Luciferase reporters containing the wild type (WT) and mutant (MUT) 3′-UTR of FGF14 that was inserted in the multiple cloning sites were constructed by Norclone (London, ON, Canada). The mutant 3′-UTR of FGF14 contained the same sequence as the WT sequence except the potential binding sequence of “ggucc” (predicted by TargetScan) was replaced with “caagg” (5 base mutation). The Dual-Luciferase reporter plasmids containing the WT 3-UTR and mutant UTR of ANG-1 or CACNA1C were constructed as well using the same method as FGF14.

H9c2 cells were placed onto a 12-well plate (80,000 cells/well) (Sigma) containing 1 mL of DMEM-CM/well and incubated for 18 h at 37 °C and 5% CO_2_. Cells were co-transfected with the constructed Dual-Luciferase reporter plasmids (0.1 μg) and miR-711 mimic or control (30 nM) using Lipofectamine 2000 at a 1:2 ratio of miRNA: Lipofectamine 2000. 48 h after transfection, cells were harvested and the luciferase assay was performed using a luciferase assay kit (Promega) according to the manufacturer’s instructions. Firefly and Renilla luciferase activity were measured using a VICTOR Multilabel Plate Reader (PerkinElmer, Woodbridge, ON) and the ratios of firefly/Renilla luciferase activity were compared. Firefly luciferase activity was measured at 560 nm, while Renilla luciferase activity was measured at 480 nm.

### Statistical analysis

All experiments were repeated at least 3 times. Data were expressed as mean ± SD. Statistical analysis was performed using paired Student’s t-test for two groups or one-way ANOVA followed by Newman-Keuls analysis for three or more groups. Statistical significance was determined at a value of *P* < 0.05.

## Results

### Oxidative stress causes over-expression of miR-711 in cardiomyocytes

**Fig. 1:**
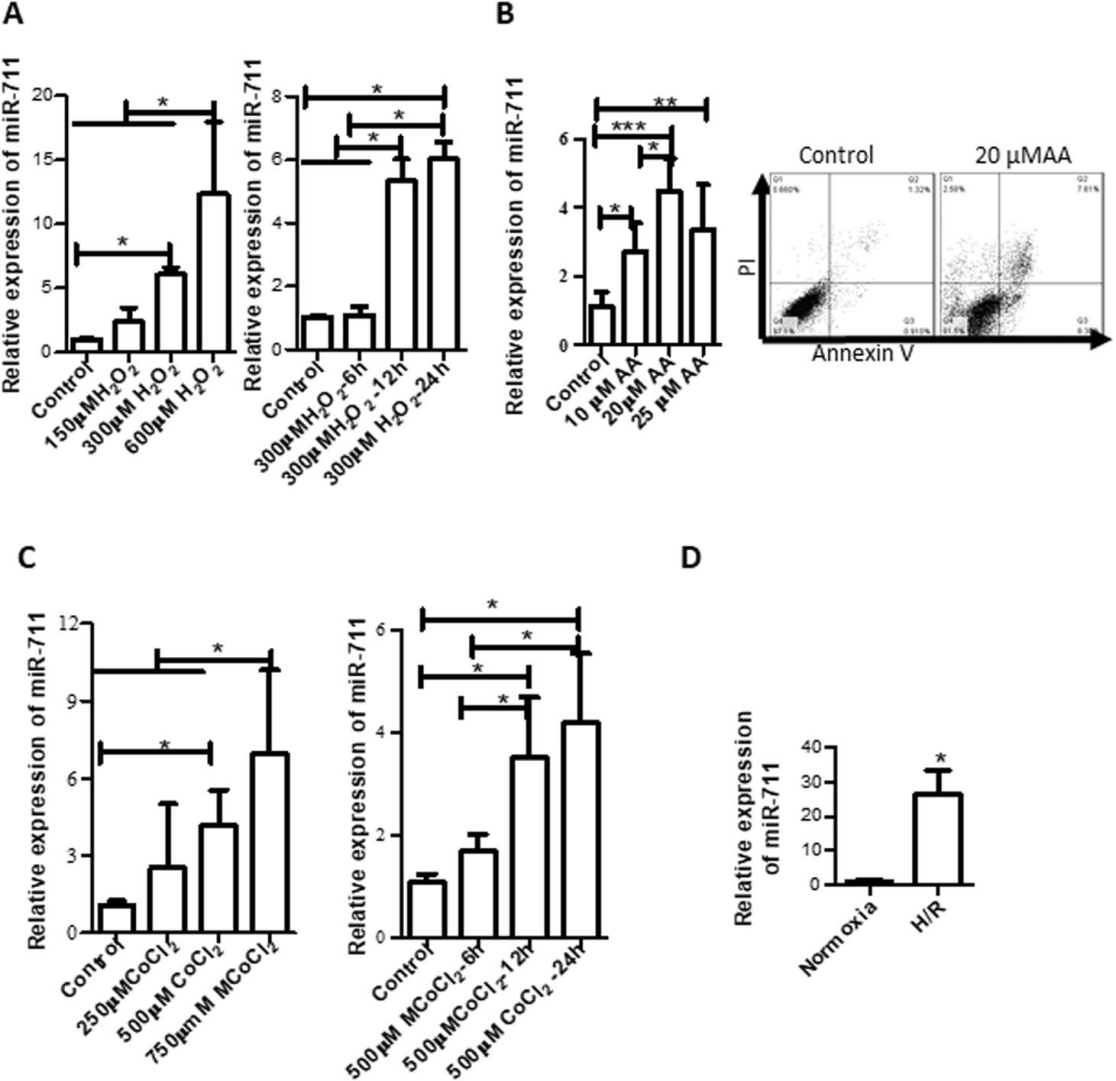
Oxidative stress up-regulated miR-711 expression. **a** H_2_O_2_ increased the expression of miR-711. H9c2 cells were cultured and treated with H_2_O_2_ at the indicated concentrations for 24 h (left panel), or with 300 μmol/L H_2_O_2_ at indicated hours (right panel). RNA was extracted using a microRNA extraction kit and miR-711 expression was detected by qRT-PCR. **b** AA induced cell apoptosis/death and up-regulated miR-711. H9c2 cells were treated with AA at the indicated concentrations for 3 h followed by culture in DMEM-CM for another 3 h. miR-711 expression was determined by RT-PCR (left panel). Cell apoptosis/death for cells treated with 20 μM AA was measured by Annexin-V and PI staining and flow cytometry (right panel). **c** CoCl_2_ treatment upregulated miR-711 expression. H9c2 cells were treated with CoCl_2_ at the indicated concentrations for 24 h (left panel), or treated with 500 μM CoCl_2_ for the indicated durations (right panel). miR-711 expression was detected by qRT-PCR. **d** Hypothermal H/R increased miR-711 expression. H9c2 cells were cultured overnight and subjected to cold hypoxia environment (0.5% O_2_ at 10 °C) for 18 h followed by 24 h reoxygenation at normal cell culture conditions. miR-711 was detected by qRT-PCR. All qRT-PCR data were presented as 2^−^∆∆Ct. Snord61 was used as a loading control. *n* = 3, *P < 0.05, ** *P* < 0.01, *** *P* < 0.05

H_2_O_2_, one of the reactive oxygen species, can cause cellular oxidative stress and cell apoptosis. We treated H9c2 cells with H_2_O_2_ at concentrations of 150 μM, 300 μM, and 600 μM for 24 h and then determined the expression of miR-711 by qRT-PCR. We found that miR-711 in cells treated with H_2_O_2_ at all tested concentrations was up-regulated compared with the untreated control (Fig. 1a left), despite the difference of miR-711 expression between 150 μM H_2_O_2_ and control was not statistically significant. We also noted that more cell death occurred as the concentration of H_2_O_2_ increased under a microscope. Additionally, we conducted a time course experiment with 300 μM H_2_O_2_. The results showed that treatment with 300 μM H_2_O_2_ for 6 h did not significantly increase the expression of miR-711, whereas both treatment for 12 h and 24 h significantly up-regulated miR-711 (Fig. 1a right). However, there was no significant difference in miR-711 expression between treatment for 12 h and 24 h.

Antimycin A (AA) is an inducer of oxidative stress and is commonly used to simulate I/R injury in the laboratory [29]. The effect of AA-induced oxidative stress on expression of miR-711 was investigated. H9c2 cells were treated with AA at concentrations of 10 μM, 20 μM and 25 μM for 3 h followed by reperfusion for 3 h. The results showed that AA at all tested concentrations significantly increased miR-711 expression in H9c2 cells compared with the control without AA treatment (Fig. 1b left panel). AA also induced cell apoptosis/death measured by double staining with Annexin-V and PI, followed by flow cytometry (Fig. 1b right panel).

CoCl_2_ is a chemical inducer of hypoxia-like responses and is commonly used to simulate I/R injury in vitro [30]. Treatment with CoCl_2_ increases ROS production and causes oxidative stress [31]. We treated H9c2 cells with 250 μM, 500 μM and 750 μM CoCl_2_ for 24 h prior to extraction of miRNA. The results showed that treatment with CoCl_2_ also dramatically up-regulated miR-711 expression (Fig. 1c, left panel). Additionally, we also conducted a time course where cells were treated with for 500 μM CoCl_2_ for 6 h, 12 h and 24 h. It showed a similar trend to H_2_O_2_, where the expression of miR-711 was significantly increased in cells treated with CoCl_2_ over 12 h compared with the untreated group (Fig. 1c, right panel). However, there was no significant increase in miR-711 expression between treatment for 12 h and for 24 h.

I/R generates ROS and induces oxidative stress. We detected the effect of I/R on miR-711 expression in H9c2 cells. H9c2 cells were cultured, exposed to a hypothermal hypoxia environment (0.5% O_2_, 10 °C for 18 h) followed by 24 h reoxygenation to mimic I/R injury in heart transplantation. miR-711 expression was detected by qRT-PCR. We found that 18 h cold hypoxia followed by 24 h reoxygenation increased the expression of miR-711 in H9c2 cells more than 6-fold compared with normoxia cells (Fig. 1d). This is consistent with our previous results from murine heart tissues and primary cardiomyocytes [21], indicating the involvement of miR-711 in H/R injury.

### Over-expression of miR-711 is responsible for cell damage under oxidative stress

**Fig. 2:**
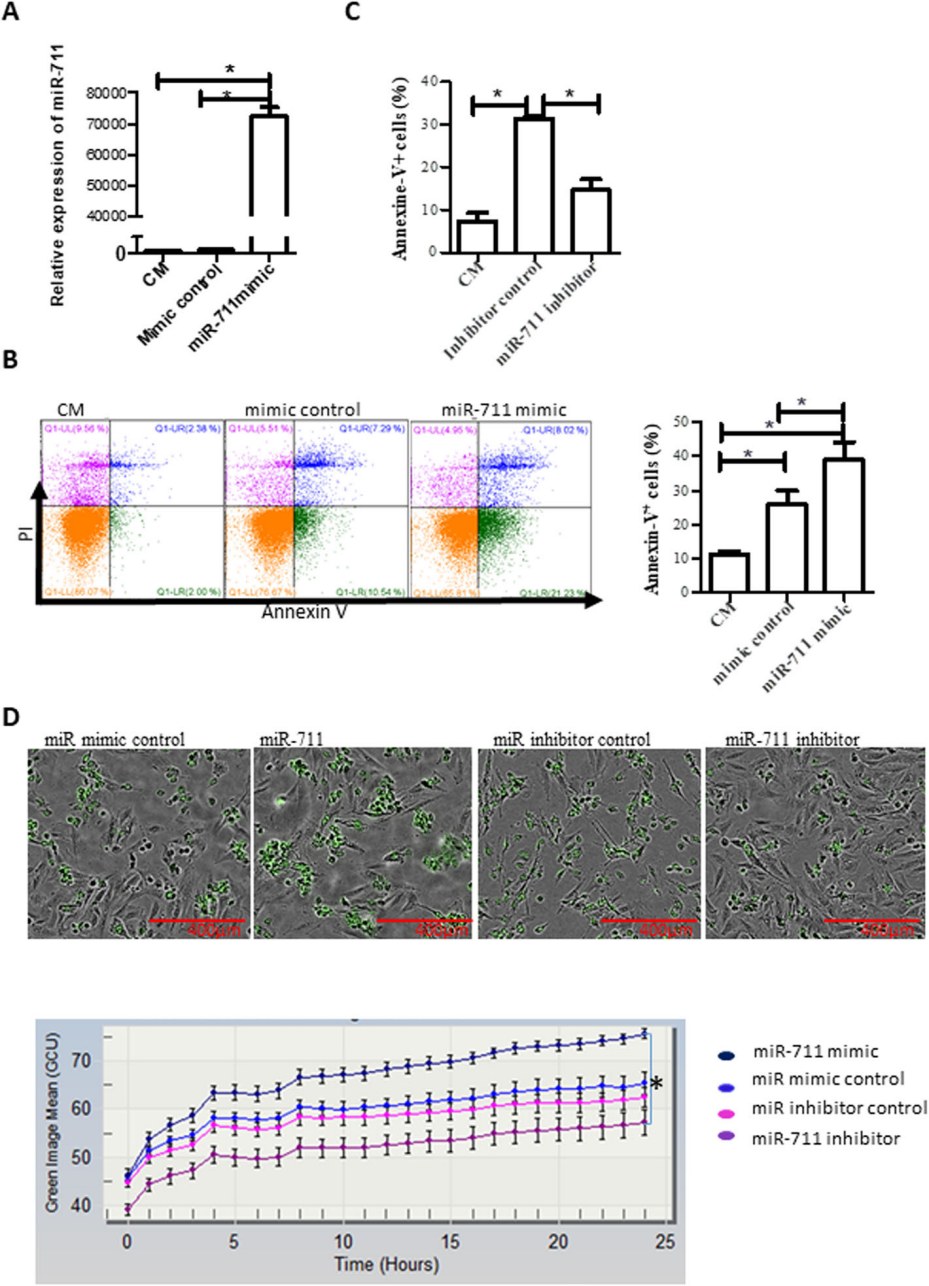
Over-expression of miR-711 increased cell damage in response to oxidative stress. **a** Transfection with miR-711 mimics increased miR-711 expression. H9c2 cells were transfected with 1 μg miR-711 mimics, or control miRNA for 48 h. miR-711 expression was detected by RT-qPCR. Snord 61 was used as an internal loading control. Data were presented using the 2^-∆∆CT^ method. **b** Over-expression of miR-711 increased cell apoptosis in response to AA. 48 h after transfection, H9c2 cells were treated with 3 h AA and 3 h reperfusion. Cell apoptosis/death was measured by Annexin-V and PI staining followed by flow cytometry. Left: Representative images of flow cytometric results; Right: Summarized data of flow cytometry from three independent experiments. **c** Inhibition of miR-711 reduced AA-induced cell apoptosis. H9c2 cells were transfected with miR-711 inhibitors 24 h prior to AA treatment. Cell death was detected as described in 2B. **d** miR-711 mimic promoted cell death induced by H/R, but miR-711 inhibitor decreased cell death, dynamically detected in an IncuCyte system. H9c2 cells were transfected with miR-711 mimic, mimic control, miR-711 inhibitor or inhibitor control for 48 h. Cells were then subjected to cold hypoxia (0.5% O_2_ at 10 °C) for 18 h. PBS was immediately replaced with DMEM-CM containing SYTOX Green dye and cells were then placed into an IncuCyte system for reoxygenation and cell death detection. Cells were scanned every hour. Images were taken at 20 h after reoxygenation (upper panel). The mean of green fluorescence per image was analyzed by the program provided by the IncuCyte system. A plot of green image means over reoxygenation time was generated (bottom panel). *n* = 3, * *P* < 0.05

The role of miR-711 in cell damage induced by oxidative stress was first studied in an AA mediated model. H9c2 cells were transfected with miR-711 mimics to increase the levels of miR-711 24 h prior to AA treatment. The levels of miR-711 were measured by qRT-PCR and cell apoptosis was determined by flow cytometry. miR-711 mimics significantly increased the levels of miR-711 (Fig. 2a) and cell apoptosis/death (Fig. 2b) as compared to control miRNA mimics. In contrast, we found that transfection with miR-711 inhibitors reduced apoptosis induced by AA (Fig. 2c).

The effects of miR-711 were also tested in an in vitro I/R model. Cells were transfected with miR-711 mimics, mimic control, miR-711 inhibitor or miR inhibitor control, prior to exposure to a cold hypoxia environment (0.5% O_2_ for 18 h at 10 °C). Cell death was detected in an IncuCyte system where SYTOX Green, which binds to DNA released from dead cells, was added to the cell medium upon reoxygenation. Cells were dynamically imaged every hour. The results showed that cells transfected with miR-711 mimics had the highest number of green fluorescent dead cells among the groups, whereas the miR-711 inhibitor transfected group had the fewest dead cells (Fig. 2d, upper panel). There was no apparent difference in cell death between the mimic control and the inhibitor control. To quantify cell death, the green fluorescent intensity over reoxygenation time was measured and calculated (Fig. 2d, bottom panel), indicating that over-expression of miR-711 increased H/R-induced cell death, but cell death was significantly attenuated by miR-711 inhibitors.

### Inhibition of miR-711 protects mitochondria from damage induced by H/R

**Fig. 3:**
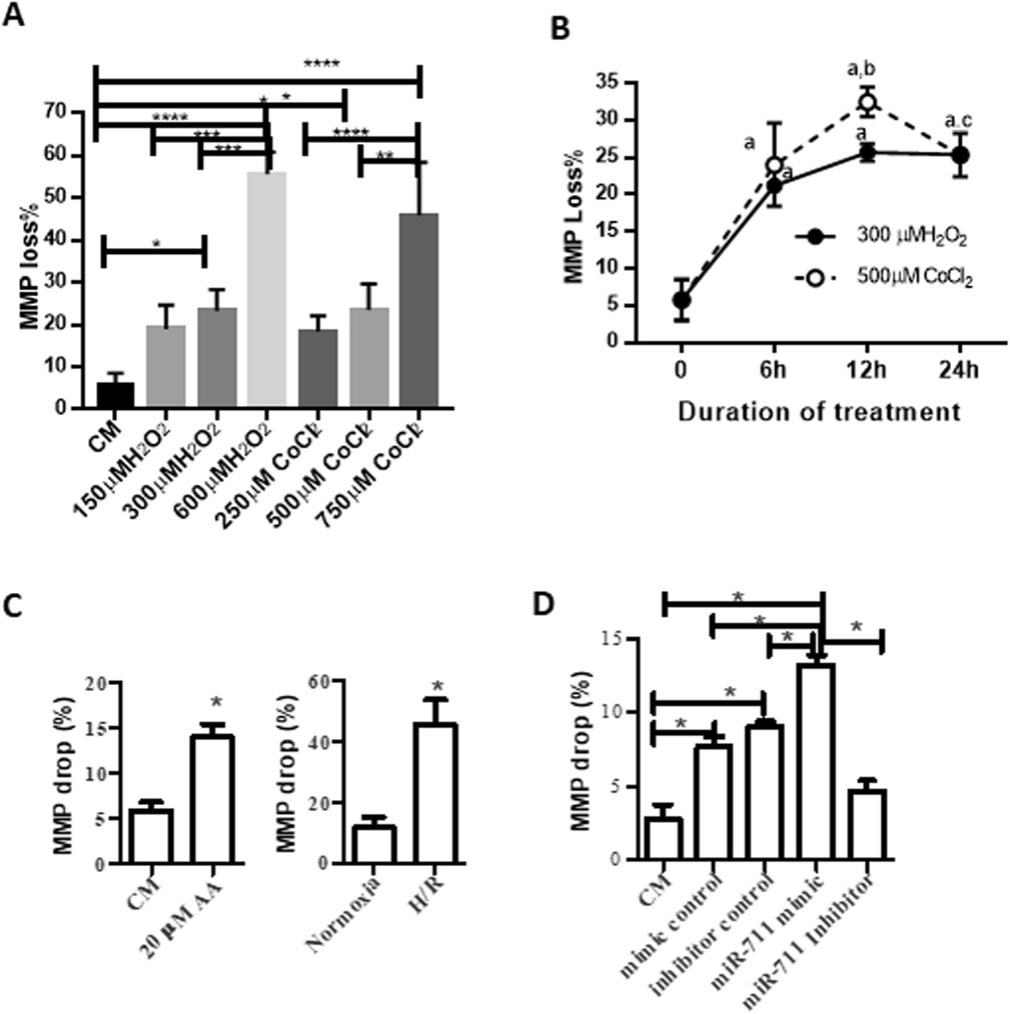
Inhibition of miR-711 reduced oxidative stress-mediated mitochondrial damage. **a** Treatment with H_2_O_2_ and CoCl_2_ decreased the MMP. Cells were treated with H_2_O_2_ and CoCl_2_ as described in Fig. 1a. Mitochondrial damage was assessed by measuring the MMP by staining cells with green fluorescent DiOC6, followed by flow cytometry. **b** The effect of treatment duration on the MMP. Cells were treated with 300 μM H_2_O_2_ and 500 μM CoCl_2_ for the indicated hours, respectively. The MMPs were detected as in (**a**). **c** AA and H/R decreased the MMP. Cells were treated 20 μM AA or exposed to cold H/R. The MMPs were detected as in (**a**). **d** Over-expression of miR-711 increased mitochondrial damage. H9c2 cells were transfected with 1 μg of miR-711 mimic or inhibitors for 48 h prior to H/R. 24 h after reoxygenation, the MMPs were measured by flow cytometry. *n* = 3, * *P* < 0.05, ** *P* < 0.01, *** *P* < 0.001, **** *P* < 0.0001. a: treatment groups compared with cells cultured in DMEM-CM; b: treatment with 500 μM CoCl_2_ for 12 h compared with treatment for 6 h; c: Treatment with 500 μM CoCl_2_ for 24 h compared with treatment for 12 h

Oxidative stress results in mitochondria damage leading to mitochondrial member potential (MMP) loss/drop [32]. To measure MMP, cells were stained with green fluorescent DiOC6 followed by flow cytometry. As shown in Fig. 3a, treatment with 300 μM H_2_O_2_, 600 μM H_2_O_2_, 500 μM CoCl_2_, or 750 μM CoCl_2_ for 24 h significantly decreased the MMP compared with the untreated group. The effects of H_2_O_2_ and CoCl_2_ on the MMP were dose dependent. In a time course experiment, we found that treatment with 300 μM H_2_O_2_, or 500 μM CoCl_2_ for 6 h started to cause a significant MMP loss, reaching the peaks at 12 h after treatment (Fig. 3b).

Both 20 AA μM and H/R also significantly increased MMP loss (Fig. 3c).

To investigate the effect of miR-711 on MMP changes, cells were transfected with miR-711 mimic, miR-711 inhibitor, or their controls 48 h prior to treatment with AA. The results showed that the MMP loss was accelerated when cells were pretreated with miR-711 mimic. By contrast, transfection with miR-711 inhibitors attenuated the MMP loss (Fig. 3d).

### miR-711 targets Ang-1, FGF14 and Cacna1c

**Fig. 4:**
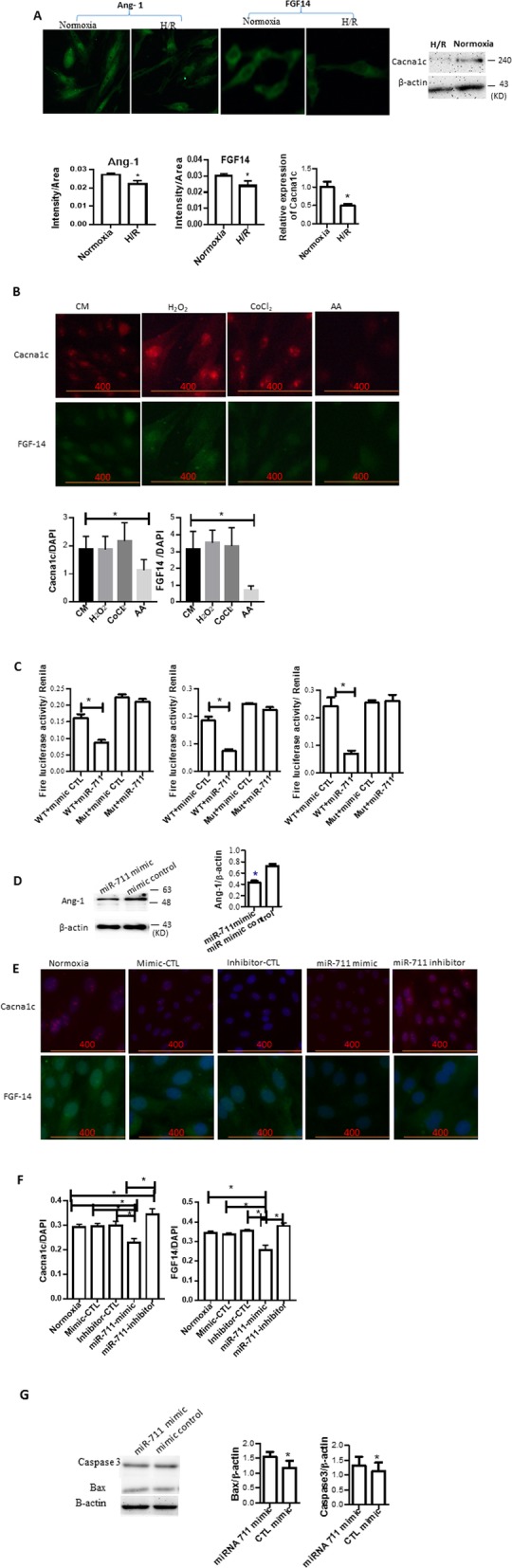
miR-711 targeted Ang-1, FGF14 and Cacna1c. **a** H/R reduced the expression of Ang-1, FGF14 and Cacna1c. Cells were treated with H/R and subjected to ICC or Western blotting with Abs against Ang-1, FGF14 and Cacna1c. Upper panel: Representative images; Bottom: Semi-quantitative results. **b** FGF14 and Cacna1c expression in cells treated with H_2_O_2_, CoCl_2_ and AA. Cells were treated for 20 μM AA for 3 h followed by 3 h reperfusion, or with 300 μM H_2_O_2_, and 500 μM CoCl_2_ for 24 h, respectively. Expression of Ang-1, FGF14 and Cacna1c was determined by Western blotting or ICC. **c** Luciferase reporter assays. H9c2 cells were transfected with firefly and Renilla dual luciferase vectors along with miR-711 mimics or miRNA mimic controls. Dual luciferase vectors contained either the wild type or mutated 3′-UTR of Ang-1, FGF14, or Cacna1c. Firefly/Renilla luciferase activities were measured 48 h after co-transfection. *n* = 3 for each group. *P < 0.05 miR-711 mimic vs. the miRNA mimic control group transfected with the same dual luciferase. WT, wild type; MUT, mutant; **d** miR-711 mimic decreased the expression of Ang-1. Cells transfected with miR-711 mimic and exposed to cold H/R. Ang-1 expression was detected by Western blotting. **e** miR-711 mimic decreased the expression of FGF14 and Cacna1c. Cells were transfected with miR-711 mimic, mimic control, miR-711 inhibitor, or inhibitor control for 48 h and then exposed to cold H/R as described in Fig. 2. Cells were fixed and subjected to ICC staining with Abs against FGF14 or Cacna1c Abs. Green: FGF14; Red: Cacna1c. **f** Quantitative results of ICC for FGF14 and Cacna1c. Fluorescent intensity of FGF14, Cacna1c and DAPI was measured using the ImageJ program. **g** Expression of Bax and Caspase 3. Cells were transfected with miR-711 48 h prior to AA treatment. Bax and caspase 3 were detected by Western blotting. β-actin was used as a loading control. *n* = 3, * *P* < 0.05

To understand the molecular mechanism by which miR-711 promotes cell apoptosis and death, we previously first performed cDNA microarray assays. The microarray results showed that Ang-1, FGF14 and Cacna1c are down-regulated in I/R injured hearts with prolonged cold ischemia in which miR-711 is over-expressed compared with control hearts [21]. We here performed immunocytochemical (ICC) staining and Western blotting to confirm their expression at the protein level in vitro. As shown in Fig. 4a, H/R with 18 h cold hypoxia significantly decreased the expression of Ang-1, FGF14 and Cacna1c compared to those in the normoxic cells (Fig. 4a).

We also detected the expression of FGF14 and Cacna1c in cells treated with 20 μM AA, 300 μM H_2_O_2_ or 500 μM CoCl_2_. AA treatment significantly reduced the expression of FGF14 and Cacna1c, whereas neither H_2_O_2_ nor 500 μM CoCl_2_ significantly changed the expression of FGF14 and Cacna2c (Fig. 4b).

Next, we performed an in silico analysis to predict potential targets of miR-711. In silico analysis indicated the presence of binding sites of miR-711 in the 3′-UTRs of Ang-1, FGF14 and Cacna1c.

Dual fluorescence reporter assays were conducted to confirm the binding of miR-711 to the 3′UTRs of the above genes. The 3′-UTR of Ang-1 was cloned into the dual fluorescence reporter plasmids. Mutant plasmids in which the seed region nucleotides were mutated were also constructed. Cells were co-transfected with plasmids containing the WT or Mutant 3′-UTR of Ang-1 and miR-711 mimic. The luciferase activities were measured using luciferase assay 48 h after transfection. As shown in Fig. 4c (left panel), the ratio of firefly/Renilla luciferase activity was significantly decreased in cells co-transfected with WT 3′-UTR plasmids and miR-711 as compared with mutant plasmids.

To validate the binding of miR-711 to FGF14 and Cacna1c, we performed experiments similar to those conducted to determine miR-711 binding to Ang-1. As expected, similar results were observed in the FGF14 and Cacna1c UTR (Fig. 4c, middle and right panels), indicating that miR-711 was able to bind to the 3′-UTR of Ang-1, FGF14, and Cacna1c.

Finally, we transfected cells with miR-711 mimic or mimic control 48 h prior to H/R treatment. Western blotting and IHC were then performed to detect Ang-1, FGF14, and Cacna1c expression, respectively, 24 h after reoxygenation. We found that transfection of miR-711 mimic significantly reduced the expression of Ang-1 (Fig. 4d), Cacna1c (Fig. 4e and f) and FGF14 (Fig. 4e and f) as compared with the mimic control. In contrast, transfection with miR-711 inhibitors ameliorated the decrease in FGF14 and Cacna1c expression induced by H/R (Fig. 4e and f).

Apoptotic genes such as Bax and caspase 3 were also detected by Western blotting. As shown in Fig. 4g, miR-711 mimic increased the expression of Bax and caspase 3.

### MiR-711 is regulated by transcription factors HIF-1a and NFКB

**Fig. 5:**
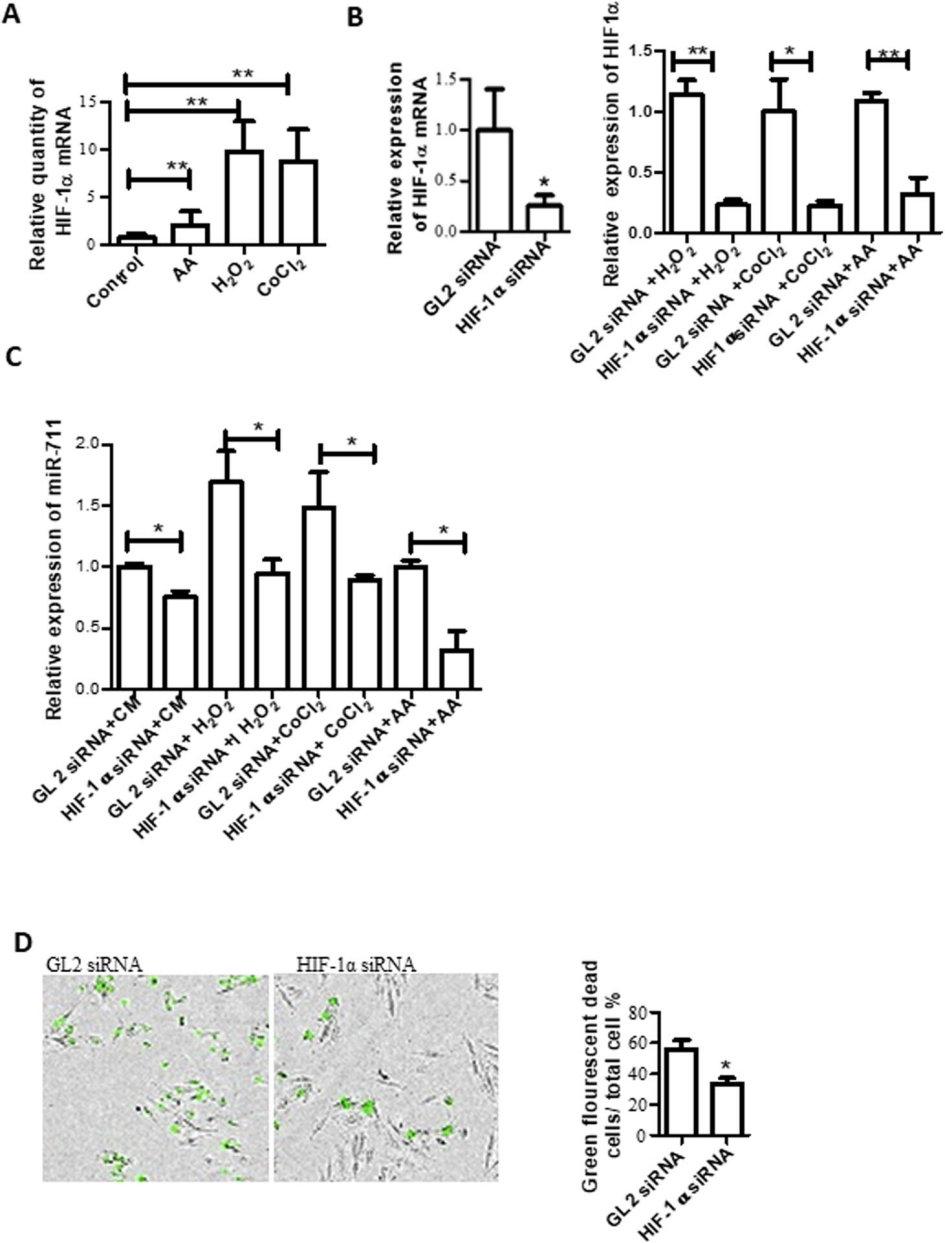
miR-711 was regulated by HIF-1α. **a** HIF-1α overexpressed by AA, H_2_O_2_ and CoCl_2_. Cells were treated with 20 μM AA, 300 μM H_2_O_2,_ and 500 μM CoCl_2_. HIF-1 α was measured by qRT-PCR. **b** HIF-1α siRNA knocked down HIF-1α. Cells were transfected with HIF-1α siRNA. 48 h after transfection, HIF-1α expression was detected by qRT-PCR (left). Some of the transfected cells were further treated with 300 μM H_2_O_2,_ and 500 μM CoCl_2_ for 24 h and HIF-1α expression was measured 24 h after treatment by qRT-PCR (right). **c** Knockdown of HIF-1α decreased miR-711 expression. MiR-711 expression was detected by qRT-PCR in the cells from (**b**). **d** HIF-1 siRNA reduced cell death induced by AA. Cells were transfected with HIF-1 siRNA for 48 h, treated with 20 μM AA for 3 h. AA was replaced with DMEM-CM containing green fluorescent DNA bound dye SYTOX and cells were placed in an IncuCyte system for real time monitoring cell death. Right: representative images of cells collected by the IncuCyte system. Dead cells appeared in green; Left: the percentage of green dead cells in total cells

To understand how miR-711 is regulated, we detected the expression of the transcription factor HIF-1a. Our results showed that AA, H_2_O_2_, or CoCl_2_ significantly increased the expression of HIF-1a detected by qRT-PCR (Fig. 5a).

To determine whether HIF-1α regulates miR-711, we transfected H9c2 cells with HIF-1α siRNA using Lipofectamine to knock down HIF-1. GL2 siRNA was used as a siRNA control. The knockdown efficacy of HIF-1α siRNA was measured 24 h after transfection. The result showed that the HIF-1α mRNA levels in cells transfected with HIF-1α siRNA were reduced by about 75% of that in control GL2 siRNA transfected cells (Fig. 5b, left panel). Next, we exposed siRNA transfected cells to oxidative stress conditions (AA, H_2_O_2_ or CoCl_2_) 24 h after transfection. The expression of HIF-1α and miR-711 was determined by qRT-PCR. We found that HIF-1α siRNA was also able to knock down the HIF-1α mRNA levels in cells treated with 20 μM AA, 300 μM H_2_O_2_ or 500 μM CoCl_2_ as compared with their GL2 siRNA controls (Fig. 5b, right panel). In normal condition without oxidative reagents, the miR-711 levels in cells transfected with HIF-1α siRNA were lower than in the GL2 siRNA control (Fig. 5c). Under oxidative stress conditions, HIF-1α siRNA significantly reduced the expression of miR-711 in cells treated with AA, H_2_O_2_, or CoCl_2_ (Fig. 5c) as compared with their control Gl2 siRNA.

In addition to miR-711 expression, we detected the effect of HIF-1 siRNA on AA-mediated cell death. The results show that there were fewer green dead cells in HIF-1α knockdown cells than in GL2 siRNA transfected cells, indicating that HIF-1α siRNA reduced cell death induced by AA (Fig. 5d).

**Fig. 6:**
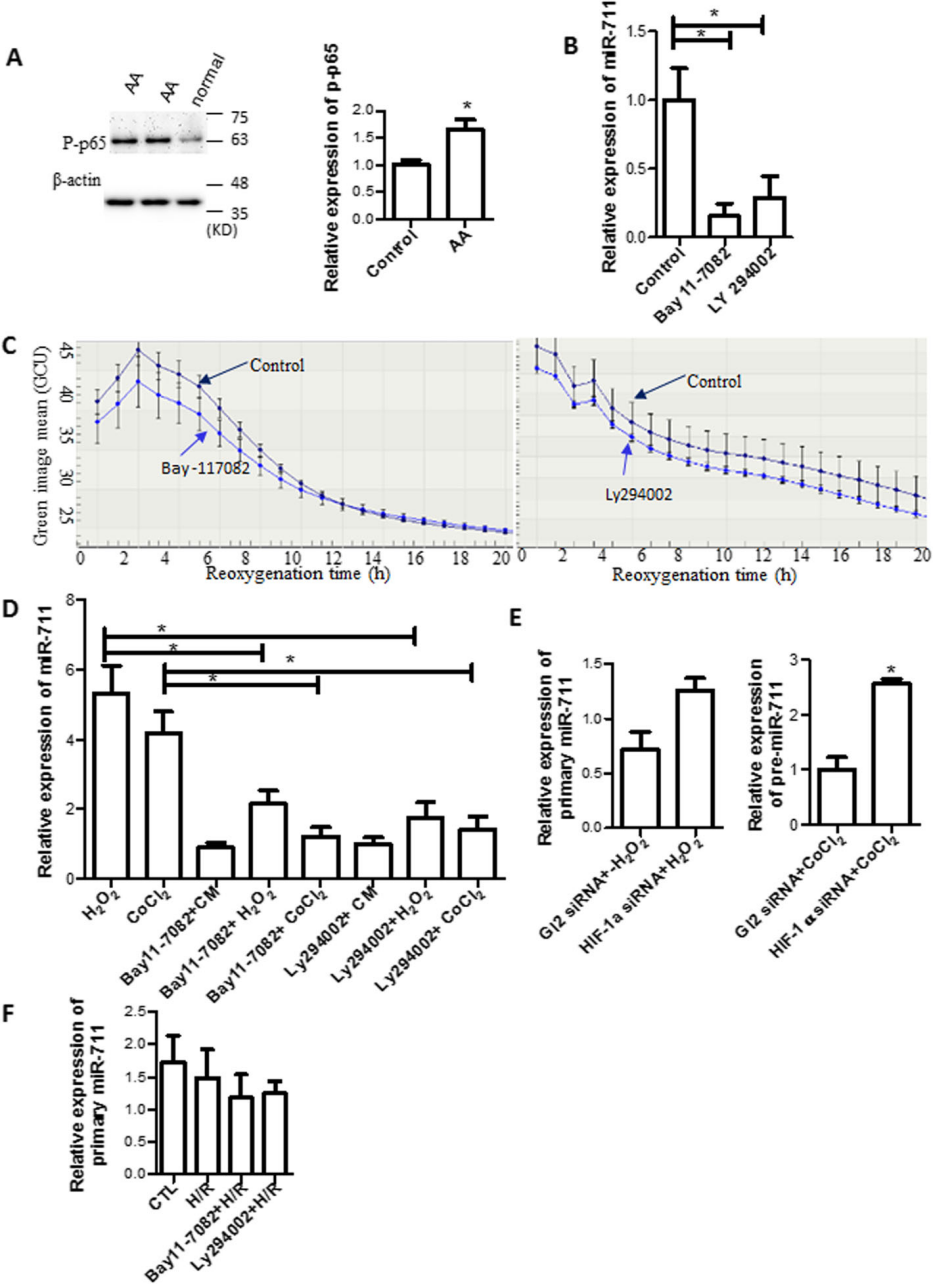
NFКB regulated miR-711 expression **a** P-p65 expression. Total protein was extracted from cells treated with AA and p-p65 expression was determined by Western blotting. Left: Representative images of Western blotting: Right: semi-quantitative results of Western blotting. **b** Inhibition of the NFКB and PI3K pathways decreased miR-711 expression. Cells were treated with NFКB inhibitor Bay 11–7082 or PI3K inhibitor LY294002 for 1 h prior to cold H/R. miR-711 was detected by qRT-PCR. **c** Treatment with Bay 11–7082 and Ly 294002 reduced cell death. Cells were pre-treated with Bay 11–7082 and LY 294002 for 1 h and then treated with H_2_O_2_ and CoCl_2_. Cell death was detected in an IncuCyte system as described in (5D). **d** Pretreatment with Bay 11–7082 and Ly 294002 reduced miR-711 induced by H_2_O_2_ and CoCl_2_. Cells were treated as described in (6C). miR-711 was measured by qRT-PCR. **e** The effect of HIF-1α siRNA on pre-miR-711 expression. Cells were treated as described in (**b**). Pre-miR-711 was measured by qRT-PCR. **f** The effect of Bay 11–7082 and Ly 294002 on Pre-miR-711. Pre-miR-711 expression was measured by qRT-PCR. *n* = 3, * *P* < 0.05

The NFКB family is an important transcription factor involved in numerous signal pathways. P65 (RelA), a member of the family, was detected by Western blotting. We found that AA treatment also increased the expression of p-p65 (Fig. 6a).

To investigate whether the activated NFКB pathway promoted miR-711 expression, cells were treated with 1 μM Bay 11–7082, a specific inhibitor of the NFКB pathway, 1 h before exposure to a hypoxic environment. As shown in Fig. 5f, the expression of miR-711 was significantly decreased in cells treated with Bay 11–7082, compared with their control cells without Bay 11–7082 treatment.

In addition, we observed that treatment with LY 294002, a specific inhibitor for phosphoinositide 3-kinase (PI3K), which is an upstream molecule of the NFКB pathway, reduced miR-711 expression (Fig. 6b). Treatment with Bay 11–7082 and Ly 294002 also attenuated cell death measured in an IncuCyte system (Fig. 6c).

We also found that treatment with Bay 11–7082 and Ly 294002 significantly reduced the expression of miR-711 induced by H_2_O_2_ and CoCl_2_ (Fig. 6d).

Furthermore, the primary miR-711 was also measured by qRT-PCR. As shown in Fig. 6e, HIF-1α siRNA did not affect pre-miR711 expression in H_2_O_2_ treated cells (left panel), but up-regulated pre-miRNA in CoCl_2_ treated cells (right panel). Neither Bay11–7082 nor Ly294002 affected pre-miR-711 expression (Fig. 6f).

## Discussion

In this study, we first demonstrated that oxidative stress as well as cold I/R upregulates miR-711 expression in heart cells. Introduction of exogenous miR-711 into cells exacerbated cell apoptosis/death and mitochondria injury, whereas inhibition of miR-711 alleviated cell injury. We demonstrated that miR-711 exacerbated cell damage induced by oxidative stresses such as H/R and AA through inhibition of the expression of Ang-1, FGF14 and Cacna1c. Furthermore, aberrant expression of miR-711 during oxidative stress is regulated by HIF-1α and the NFКB signaling pathway.

Studies have shown that miR-711 is up-regulated in trauma injured brains and heart cells with warm H/R injury and that over-expression of miR-711 causes cell death [22–25]. Additionally, miR-711 is associated with HIV infection [26] and tumor growth [27, 28]. We previously showed that miR-711 is dramatically up-regulated in hearts with I/R injury occurring in heart transplantation and H/R injured primary cardiomyocytes isolated from neonatal mice [21]. In this study, we found that oxidative stress caused by H_2_O_2_, CoCl_2_, AA and hypothermal H/R significantly up-regulated miR-711 while increasing cell death and mitochondrial damage as evidenced by apparent cell morphological changes (loss of spindle shape, decrease in size, and existence of cell debris) and MMP loss. The effects of H_2_O_2_, CoCl_2_, and AA on miR-711 expression, cell death and mitochondrial damage were dose dependent. A significant increase in miR-711 was observed at 12 h after treatment with 300 μM H_2_O_2_ and 500 μM CoCl_2_, demonstrated by time course experiments. Moreover, transfection of miR-711 mimics before induction of oxidative stress such as treatment with AA or exposure to H/R conditions accelerated cell damage. In contrast, transfection of miR-711 inhibitors reduced cell injury.

miRNA negatively regulates gene expression through partial base pairing to the 3′-UTRs of its target mRNAs, inhibiting its target gene protein translation or destabilizing mRNA. A single miRNA is capable of inhibiting multiple genes. Previous studies have demonstrated that miR-711 targets Ang-1 and AKT with sequential inactivation of anti-apoptosis genes, resulting in apoptosis/death of neuronal cells [22, 24]. Zhao et al. also reported that miR-711 targets calnexin in rat cardiomyocytes, thus leading to endoplasmic reticulum stress-mediated apoptosis after myocardial infarction [25]. Yang Q et al. also reported that miR-711 targets Notch1 in HIV-1 in PEL cell lines [26]. In this study, we confirmed that miR-711 targeted Ang-1, which was downregulated in I/R injured or oxidative stressed cells, leading to an increase in the expression of apoptotic genes caspase 3 and Bax. Lee et al. previously demonstrated that Ang-1 can exert cardioprotective effects by preventing vascular leakage and cardiomyocyte death through inhibiting activities of caspase 3 and caspase 9 [33]. This suggests that the downregulation of Ang-1 expression by miR-711 could enhance the damage caused by H/R injury. Moreover, we observed that miR-711 targeted the *FGF14* and *Cacn1c* genes, which were down-regulated in cells treated with H/R and AA. FGF14 is a member of the fibroblast growth factor (FGF) family, which is heavily involved in cell growth and tissue repair. Although there have been no direct reports related to FGF14 and cardiac cell death, data from neuron cell studies showed that FGF14 is associated with cell apoptosis [34] and that a deficiency of FGF14 resulted in cell death [35]. This implies that FGF14 plays a role in cell apoptosis. Cacna1c, also known as Cav1.2, is a subunit of the L-type voltage-dependent calcium channel. Calcium channels mediate the influx of calcium ions into the cell and are involved in a variety of calcium-dependent processes, including cell division and cell death. Boczek et al. reported that homozygous knock-out of the *Cacna1c* gene is lethal in mice and downregulation of Cacna1c increases p38MAPK expression [36]. In this study, we observed decreased levels of Cacna1c accompanied by a profound increase of p38MAPK in H/R injured and oxidative stressed cells. This implies that there may be an interaction between Cacna1c downregulation, p38MAPK and cell death in heart cells as well. Further studies need to be conducted in order to confirm this relationship. Additionally, we observed that pre-treatment with miR-711 mimic increased the expression of the apoptotic genes caspase 3 and Bax in response to AA stress. Taken together, our data suggest that oxidative stress up-regulates miR-711, resulting in the reduction of Ang-1, FGF14 and *Cacn1c*, leading to over-expression of apoptotic genes caspase 3 and Bax, subsequently induces cell apoptosis/death in response to AA and H/R.

It is unexpected that H_2_O_2_ or CoCl_2_ did not significantly change the expression of FGF14 and Cacna1c. In contrast, we noted that treatment with H_2_O_2_ or CoCl_2_ enhanced aggregation of Cacna1c in the nucleus. These results imply that there might be other molecules in addition to miR-711 that regulate FGF14 and Cacna1c. Other known molecules might dampen the effect of miR-711 on the above two proteins. It is also possible that miR-711 does not target these two molecules because one miRNA could have multiple targets and its effect is dynamic. More potential targets of miR-711 need to be investigated in future to better understand how miR-711 influences cells in response to H_2_O_2_ or CoCl_2_.

miRNA is non-coding RNA transcribed by RNA polymerase II. Its biogenesis is temporally and spatially regulated by multiple factors including transcription factors and epigenetic modification [37]. In this study, we focused on the two highly expressed transcription factors HIF-1α and NFКB, in response to stress and their roles in regulating miR-711. HIF-1α is a main regulator of gene expression during hypoxic stress and plays dual roles in the heart in response to stress: cardioprotective and cardiodeleterious [38]. HIF-1α has been shown to regulate P53 and BN1P3 genes, leading to induction of apoptosis and mitophagy [39]. In this study, we found that oxidative stress induced HIF-1α, which further promoted miR-711 expression, resulting in cell death. In contrast, inhibition of HIF-1α led to a reduction in both miR-711 expression and cell death in response to oxidative stress. Our results indicate that HIF-1α plays a role in upregulation of miR-711. This is a novel finding as the upstream regulatory pathway of miR-711 expression during I/R was not known previously.

NFКB is a transcription factor which transcribes both survival and apoptotic genes. Both protective and causative effects of NFКB signaling on heart cells with I/R or H/R injury have been observed. It has also been reported that activation of NFКB or PI3K by stress increases miRNA expression [40]. Our results showed that oxidative stress activated the NFКB pathway, which corroborates the findings of Zhang J et al. suggesting that warm hypoxia reperfusion increased the expression of NFКB [23]. We also found that inhibition of the pathway with low concentrations of Bay 11–7082 and its upstream PI3K inhibitor Ly294002 reduced cell death and miR-711 expression. The data suggest that the NFКB pathway positively regulates miR-711 biogenesis. However, we noted that a higher concentration of Bay 11–7082, for example, 10 μM, caused cell death and up-regulated miR-711.

We also found that inhibition of HIF-1α did not change the expression of pre- miR-711 in H_2_O_2_-treated cells, but increased pre-miR-711 in CoCl_2_-treated cells. This suggests that HIF-1α may not only affect the biogenesis of miR-711 but also influence degradation of miRNA in a stress model dependent mode.

Both Bay 11–7082 and Ly294002 reduced pre-miR-711 expression, but it did not reach statistical significance. Therefore, those transcription factors possibly affect transcription of miRNA as well as miRNA catabolism.

This study is focused on heart cell line H9C2 cells, but not on other heart cells, or primary cardiomyocytes, which is one of the limitations of the study. It needs to be tested on other heart cells in future.

## Conclusions

In conclusion, our results indicate that oxidative stresses (AA, H_2_O_2_, CoCl_2_ and H/R) dramatically increased the expression of miR-711 in heart cells. Over-expression of miR-711 increased cell apoptosis/death and mitochondrial damage in response to oxidative stress. miR-711 negatively regulates Ang-1, FGF14 and Cacna1c in response to AA and H/R. Both HIF-1α and NFКB regulate miR-711 expression under oxidative stress in H9c2 cells. miR-711 might be a potential new target in the prevention of damage induced by oxidative stress and I/R injury.

## Data Availability

All data generated or analyzed during this study are included in this manuscript.

## Abbreviations

AA: antimycin A
Ang-1: angiopoietin 1
Cacna1c: calcium voltage-gated channel subunit alpha1C
FGF14: fibroblast growth factor 14
H/R: hypoxia/reoxygenation
HIF-1α: hypoxia inducible factor 1a
I/R: ischemia reperfusion
ICC: in situ immunocytochemical staining
miR-711: microRNA 711
miRNA: microRNA
MUT: mutant
NFКB: nuclear factor kappa-light-chain-enhancer of activated B cells
p38MAPK: P38 mitogen-activated protein kinases
PI: propidium iodide
PI3K: phosphoinositide 3-kinases
p-P65: phosporylated-p65
PPARγ: peroxisome proliferator-activated receptor gamma
PVDF: polyvinylidene difluoride
qRT-PCR: quantitative reverse transcriptase-polymerase chain reaction
RIPA: radio immunoprecipitation assay
siRNA: small interference RNA
TBS: Tris-buffered saline
TBST: Tris-buffered saline (TBS) and Tween 20
UTR: untranslated region
WT: wild type
